# Neuronal RNA processing: cross-talk between transcriptional regulation and RNA-binding proteins

**DOI:** 10.3389/fnmol.2024.1426410

**Published:** 2024-08-01

**Authors:** Hasan Can Ozbulut, Valérie Hilgers

**Affiliations:** ^1^Max-Planck-Institute of Immunobiology and Epigenetics, Freiburg, Germany; ^2^Faculty of Biology, Albert Ludwig University, Freiburg, Germany; ^3^International Max Planck Research School for Immunobiology, Epigenetics, and Metabolism (IMPRS-IEM), Freiburg, Germany

**Keywords:** neuronal RNA processing, transcription factors, RNA-binding proteins, alternative polyadenylation, RNA, nervous system

## Abstract

In the nervous system, alternative RNA processing is particularly prevalent, which results in the expression of thousands of transcript variants found in no other tissue. Neuron-specific RNA-binding proteins co-transcriptionally regulate alternative splicing, alternative polyadenylation, and RNA editing, thereby shaping the RNA identity of nervous system cells. Recent evidence suggests that interactions between RNA-binding proteins and cis-regulatory elements such as promoters and enhancers play a role in the determination of neuron-specific expression profiles. Here, we discuss possible mechanisms through which transcription and RNA processing cross-talk to generate the uniquely complex neuronal transcriptome, with a focus on alternative 3′-end formation.

## Introduction

Neurons are structurally and functionally complex cells that constantly adapt to their environment and to external stimuli. This necessitates a rapid, dynamic yet robust coordination of gene expression, a task that neurons achieve by specifically modulating transcription and RNA processing. Alternative splicing (AS) and alternative polyadenylation (APA) of mRNA precursors can generate multiple mRNA isoforms from the same transcription unit. In APA, the use of several functional polyadenylation [poly(A)] sites results in mRNA isoforms with different 3′-ends. When alternative [poly(A)] sites are located upstream of the stop codon, transcripts differ in their protein coding potential. More commonly, mRNAs with different 3′ UTRs are generated (Mitschka and Mayr, 2022). Notably, in animals from flies to humans, hundreds of genes undergo a shift toward more distal [poly(A)] sites exclusively in neurons, thus producing longer, often ultra-long, 3′ UTRs, termed “neuronal 3′ UTRs” (nUTRs) (Hilgers et al., 2011; Smibert et al., 2012; Miura et al., 2013; Carrasco et al., 2020; Wei et al., 2020). The alternative use of splice sites through AS is also particularly prevalent in neurons; the selective inclusion or exclusion of exons results in thousands of neuron-specific transcript variants (Carrasco et al., 2020; Lee et al., 2021). One particularly striking example of neural-regulated AS is the systematic inclusion of <30 nucleotide “microexons” (Irimia et al., 2014) that is mediated by the eMIC protein domain across Bilateria (Torres-Méndez et al., 2019). Interestingly, the splicing programs independently evolved in nonvertebrate and vertebrate bilaterians, but ultimately regulate neuronal excitability: in mammals, neuronal microexons encode amino acids on the surface of interaction domains of proteins involved in neurogenesis, whereas in flies, top splicing targets are enriched in ion channels (Torres-Méndez et al., 2022).

An integral and conserved feature of neurogenesis, neuronal RNA processing generates mRNA isoforms that differ in their coding or UTR sequence, thereby increasing proteome diversity and fine-tuning gene expression [reviewed in (Bhat et al., 2022, Hilgers, 2022, Wei and Lai, 2022, Lee et al., 2023)]. Neuron-specific RNA isoforms play an important role in neurogenesis (Zhang et al., 2019; Bae et al., 2020; Bae and Miura, 2020; Carrasco et al., 2020) and contribute to the versatility of neuronal cells by helping coordinate specialized processes. Although the importance of RNA-based regulation in human neurological disease has been known for decades, the underlying pathogenic mechanisms are still not well understood.

## RNA-binding proteins regulate alternative RNA processing in neurons

RNA processing is regulated by a myriad of RNA-binding proteins (RBPs) that usually act in a cell-, gene-, and context-specific manner. Many RBPs are enriched or exclusively expressed in neural tissues, and consequently mediate RNA processing in a nervous-system-specific manner. Such RBPs and their molecular roles are typically well-conserved across metazoans; they include members of the protein families ELAV (Embryonic Lethal Abnormal Vision)/Hu (Human antigen) PTBP (Polypyrimidine Tract-Binding Protein), NOVA antigen (Neuro-oncological ventral), RBFOX (RNA-binding Fox-1 homolog), and CELF (CUGBP Elav-like family). The role of these protein families in neuronal RNA processing have been recently described in an excellent review (Lee et al., 2023). In this perspective article, we will maintain a focus on the well-studied protein ELAV as a representative model for neuron-specific RBPs and their interactions with transcriptional processing.

ELAV/Hu proteins highly conserved RBPs critical for neuronal differentiation, maturation and function (Mirisis and Carew, 2019; Hilgers, 2022; Wei and Lai, 2022; Mulligan and Bicknell, 2023). Typically, at least one member of the ELAV/Hu protein family is expressed specifically in neurons, and ELAV/Hu proteins serve as markers for neuronal cell types throughout the animal kingdom (Pascale et al., 2008). In Drosophila, where it was first described, ELAV regulates AS as well as APA (Koushika et al., 1996, 2000; Soller and White, 2003). Genome-wide studies in Drosophila revealed that ELAV/Hu operate on the transcriptome scale, with hundreds of genes undergoing ELAV-dependent alternative processing (Hilgers et al., 2012; Carrasco et al., 2020; Wei et al., 2020; Lee et al., 2021). Strikingly, all neuron-specific APA events were shown to depend on ELAV; the RNA signatures mediated by ELAV/Hu proteins are so manifold and distinct that the RBP is considered a “master regulator” of neuronal RNA processing in Drosophila. It remains to be seen whether ELAV/Hu proteins possess a similar monopoly in other systems; evidence from individual genes in human and mouse systems suggests that they do (Zhu et al., 2007; Dai et al., 2012; Mansfield and Keene, 2012; Dorrity et al., 2023), although the molecular intricacies remain to be solved. In Drosophila, ELAV binds nascent transcripts in the vicinity of proximal poly(A) and splice sites to inhibit their usage and foster APA and AS, respectively. Nearly all mRNAs found to be deregulated in *elav* mutants were direct binding targets of ELAV as seen by iCLIP in fly brains (Carrasco et al., 2020), suggesting that ELAV regulates its functional APA targets through direct physical interaction. In contrast, while ELAV binds to many AS targets at relevant splice sites (Carrasco et al., 2020; Lee et al., 2021), indicative of a direct effect, an indirect role was also described: ELAV mediates neuronal APA of *Srrm234*, and the resulting eMIC-containing isoform of Srrm234 in turn globally promotes the inclusion of neural microexons (Torres-Méndez et al., 2022).

## A role for cis-regulatory elements in alternative RNA processing

In addition to *trans*-acting factors such as RBPs, recent findings point to a role for cis-regulatory sequences —promoters, enhancers— and their associated effectors —transcription factors— in the regulation of RNA processing. Early studies showed a physical association of RNA processing factors with the transcription machinery, as well as a positive influence of transcriptional activation on 3′-end processing (Dantonel et al., 1997; Calvo and Manley, 2003; Glover-Cutter et al., 2008; Wang et al., 2010; Nagaike et al., 2011; Yang et al., 2016; Carminati et al., 2023). Conversely, effective co-transcriptional processing is necessary for RNA Polymerase II (Pol II) processivity (Tellier et al., 2020). The mechanistic underpinnings of the regulatory coupling between transcription initiation, processing and transcription termination are not well understood. Accumulating evidence has shown that these couplings are important for context-, tissue-, and gene-specific APA and AS. Correlations between the use of distinct transcription start sites (TSSs) and 3′-end processing at different poly(A) sites have been observed, for example in different cell types (Anvar et al., 2018; Hardwick et al., 2022) and in the disease context (Demircioğlu et al., 2019). Recent studies have now established a causal link between sites of transcription initiation and sites of RNA processing: in Drosophila brains and human cerebral organoids, specific TSSs —so-called “dominant promoters”— foster the selection of distinct splice and 3′-end processing sites. Promoter dominance is highly prevalent in the nervous system, occurring in about 40–60% of genes, and broadly regulates mRNA isoform selection (Alfonso-Gonzalez et al., 2023). A role for distal gene enhancers and the relative position of the TSS relative to 3′-end sites on the DNA template have also been shown to influence 3′-end processing activity, and consequently, the expression of alternative 3′ UTR isoforms (Kwon et al., 2022; Calvo-Roitberg et al., 2024). These couplings between transcription initiation and RNA processing choices suggest a widespread coordination of events that occur during transcription; they also imply that many RNA processing events are regulated as soon as transcription initiates, many kilobases upstream.

### Regulation of RNA processing by transcription factors

Transcription factors (TFs), the key effectors and regulators of transcription, likely play an important role in coordinating transcription initiation and RNA processing. While it is commonly understood that they primarily function at the chromatin level by binding directly to DNA, it is less recognized that a subset of TFs, termed DRBPs (DNA- and RNA-binding proteins), also have the capability to bind RNA.

For example, the Hox transcription factor Ultrabithorax (Ubx) binds to nascent pre-mRNAs at alternative cassette exons through its homeodomain, thereby promoting exon inclusion in the Drosophila mesoderm (Carnesecchi et al., 2022). Interestingly, Ubx interacts with chromatin in a dynamic, transcription elongation-dependent manner, indicating that Ubx may accompany Pol II from initiation to processing using different nucleic acid binding modules or assembling distinct functional complexes “on the go.”

A potentially widespread function for TFs in AS arose from studies in which knockdown of TFs with C2H2-type zinc finger (ZnF) DNA-binding domains had pronounced effects on splicing events in K562 and mouse neural cells (Han et al., 2017; Ullah et al., 2023). For at least a subset of ZnF TFs, the modulation of exon inclusion/exclusion and intron retention seemed to occur through direct binding of nascent RNA at intronic regions. One such ZnF, Zfp871, regulates hundreds of neural-differential exons in genes typically associated with neuronal morphology and function, hinting at a broad role for ZnF TFs in the regulation of neuron-specific RNA processing (Han et al., 2017).

Recent findings also reveal an involvement of the transcriptional co-activators CREB-binding protein (CBP)/p300 in alternative 3′-end site selection. In Drosophila brains, CBP was found enriched at dominant promoters as well as at their associated, usually distal, 3′-end site. Strikingly, genetic deletion of CBP resulted in a broad disruption of the 3′-end expression landscape in developing embryos (Alfonso-Gonzalez et al., 2023). How CBP connects sites of transcription initiation and alternative processing, remains unknown; given the essential role of CBP in neuronal differentiation (Lipinski et al., 2019), understanding this interaction could provide clues into the promoter-mediated establishment and maintenance of the neuron-specific 3′-end landscape.

A recent, genome-wide study found that nearly half of all TFs can bind RNA in human cells. Interactions occur through a novel, highly conserved arginine-rich motif (ARM) and were shown to enhance the TF’s association with chromatin, thereby promoting target gene expression (Oksuz et al., 2023). Missense mutations in ARM motifs were associated with human diseases, including cancer and developmental syndromes; perturbations of key TF’s ARMs without affecting DNA binding resulted in developmental defects in zebrafish, which suggests that RNA binding constitutes a widespread property of TFs that contribute to their function *in vivo*.

In contrast, several recent studies report that multiple chromatin proteins previously described as DRBPs, including PRC2, JARID2, and YY1, do not appear to bind RNA *in vivo*. PRC2 core subunits did not associate with RNA under stringent experimental conditions (Guo et al., 2024); the loss of PRC2 enrichment at chromatin upon RNase treatment can be explained, at least in part, by a concomitant, unspecific enrichment of non-target regions (Hall Hickman and Jenner, 2024; Healy et al., 2024). It will be important to verify the actual binding of TFs to RNA on a case-by-case basis in order to distinguish direct and indirect effects on the regulation of RNA metabolism (Nielsen and Ulitksy, 2024). Although many protein-RNA interactions remain to be functionally validated and mechanisms to be elucidated, chromatin proteins have emerged as key players in the regulation of RNA processing.

### Interaction of RBPs with cis-regulatory elements

The connection between RNA processing and transcription regulation in cis is supported by the widespread occurrence of physical and genetic interactions between splicing/ polyadenylation factors and the transcription machinery [reviewed in Bentley (2014), Shenasa and Bentley (2023), and Shine et al. (2024)]. In addition to an effect of RBPs on Pol II processivity through their interaction with transcribing RNA, multiple lines of evidence also indicate that RBPs interact with chromatin to regulate promoter activity in a promoter- and gene-specific manner. Several genome-wide studies suggest that many nuclear RBPs exert their function at the chromatin level. By ChIP-seq, RBPs were found to pervasively, extensively, and specifically associate with DNA at gene promoters (Xiao et al., 2019); in another study, RBPs even constituted nearly half of all proteins obtained from the chromatome (Rafiee et al., 2021). One RBP with a demonstrated role at gene promoters is the splicing factor Rbm25, which co-associates with the TF YY1 at numerous genetic loci; the physical interaction between the two proteins is necessary for YY1 recruitment to chromatin and transcriptional output (Xiao et al., 2019), suggesting a role of RBPs in the regulation of promoter activity. Whether RBPs are recruited to the DNA template via RNA binding, through the RBP’s intrinsically disordered regions (IDRs), or through chromatin-associated proteins, likely differs on a gene- and RBP-dependent basis.

The first evidence of promoter sequences in the regulation of APA was shown in the context of neuron-specific RNA processing. In the Drosophila nervous system, the RBP ELAV physically associates with the promoters of genes that undergo ELAV-dependent APA and 3′ UTR lengthening. Selection of the distal, neuron-specific 3′-end site was abrogated upon replacing the native promoter of an ELAV target gene by a generic one; moreover, ectopic ELAV expression in muscle cells induced neuronal 3′ UTRs from transgenes carrying the native, but not the generic promoter. The ELAV binding pattern coincided with the signature of Pol II promoter-proximal pausing, indicating that ELAV may be loaded onto the transcription machinery during transcription initiation (Oktaba et al., 2015). It remains unclear how ELAV then finds its way to its functional sites on the nascent RNA —proximal poly(A) sites potentially located kilobases further downstream (Hilgers, 2015; Slobodin and Agami, 2015).

The RNA and DNA-binding protein Fused in Sarcoma (FUS), linked to amyotrophic lateral sclerosis (ALS) (Kwiatkowski et al., 2009; Vance et al., 2009), functions in multiple RNA processes in neuronal cell nuclei. FUS was shown to co-transcriptionally binds to pre-mRNAs to regulate AS (Ishigaki et al., 2012; Lagier-Tourenne et al., 2012); FUS iCLIP clusters on nascent RNA positionally coincide with RNA Pol II pausing sites (Masuda et al., 2015). Moreover, *in vitro* experiments indicate that FUS mediates the physical and functional interactions between the transcription and splicing machineries (Yu and Reed, 2015). Interestingly, the histone mark H3K36me3 in actively elongating genes was recently shown to recruit FUS to chromatin and away from nascent RNA, thereby ensuring proper poly(A) site selection (Jia et al., 2024).

As more examples arise of transcription factors and cis-regulatory elements that control RBP-mediated AS and APA, it will be interesting to determine whether common mechanisms govern these interactions, or if they differ based on the gene and cellular context.

## Possible mechanisms linking transcription to co-transcriptional processing

Several scenarios can be envisaged to explain how RBPs interact with gene activation and transcription processes, regulating AS and APA in a tissue-specific manner. Although our hypotheses are formulated with neuronal RBPs in mind, the proposed mechanisms are not mutually exclusive, and each of them may operate in different contexts or tissues.

**Figure 1:**
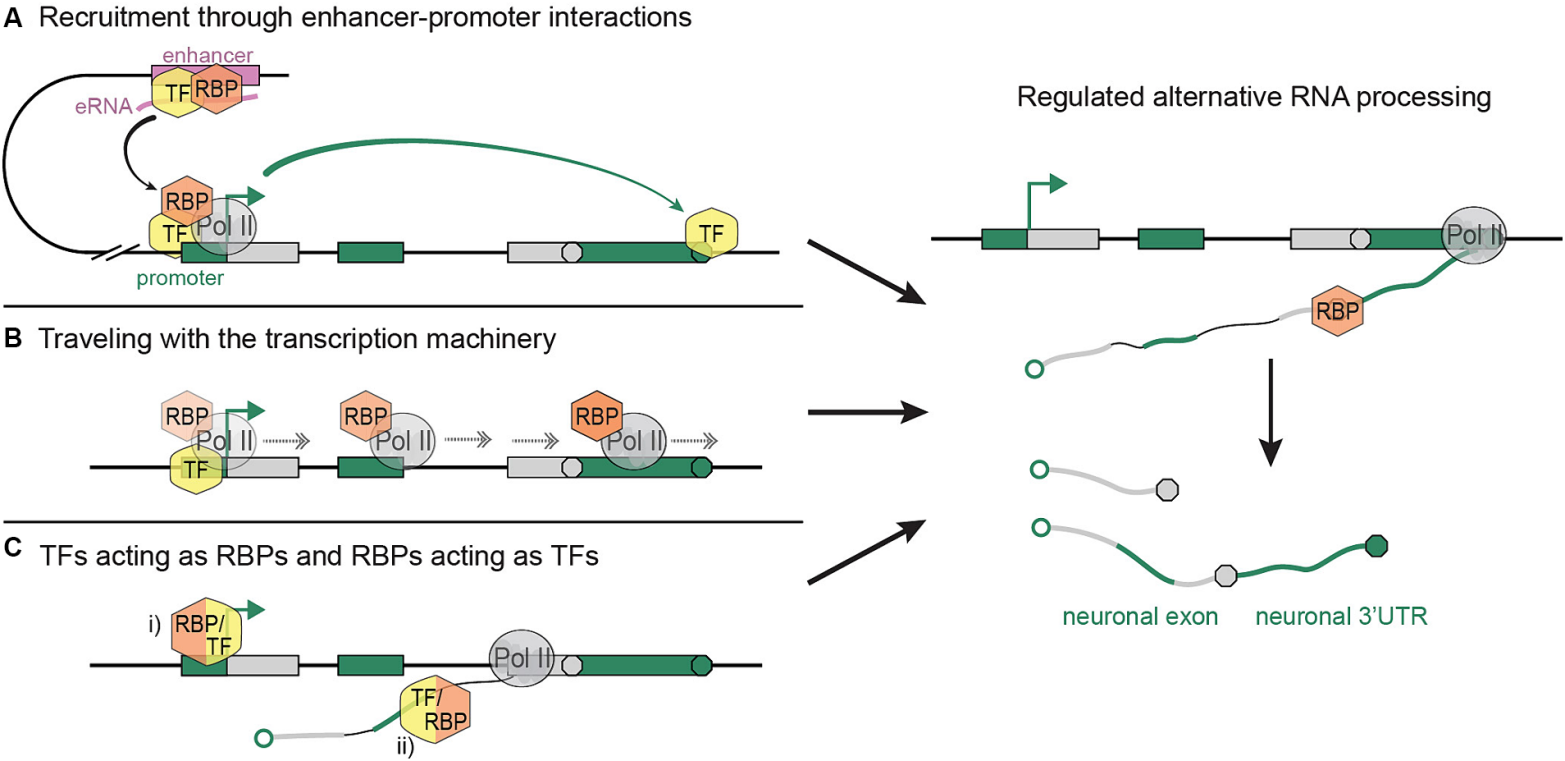
Possible mechanisms linking cis- and trans-regulation of tissue-specific RNA processing. **(A)** RBPs are recruited to tissue-specific enhancers and their target promoter through binding to TFs and/or enhancer RNAs. The activation of a dominant promoter fosters the expression of the linked, tissue-specific 3′-end. **(B)** RBPs associate with the transcription machinery at gene promoters by binding to TFs or the Pol II CTD, and accompany the elongating transcription complex to downstream sites of alternative RNA processing. **(C)** Intertwined regulation of transcription and RNA processing through (i) RBPs acting as TFs at the gene promoter, and/or (ii) TFs acting as RBPs on nascent pre-mRNAs. Resulting tissue-specific (neuronal) RNA processing events are represented on the right. Sequences that are expressed in a tissue-specific (neuronal) fashion are shown as green boxes (DNA) and lines (RNA).

Interestingly, enhancer regions help modulate 3′-end processing choices (Kwon et al., 2022; Calvo-Roitberg et al., 2024); it is conceivable that RNA processing factors are recruited to specific genes through enhancer-promoter interactions and the TFs that mediate them. One possible mechanism is exemplified by the histone acetyltransferase and chromatin remodeler CBP, which binds RNAs transcribed from enhancer regions (eRNAs) and stimulates transcription at target promoters (Bose et al., 2017). CBP binds both to neuron-specific TSSs and associated, often neuron-specific 3′-end sites (Alfonso-Gonzalez et al., 2023), thereby creating a link between the spatiotemporal regulation of gene activation and RNA processing. TFs like CBP may guide RBPs to gene promoters by first promoting recruitment to enhancer regions through direct (TF-RBP) or indirect (TF-eRNA-RBP) interactions (Figure 1A).

Transcriptional processivity and elongation speed are essential for proper RNA processing, especially in long genes; Pol II slowing, pausing, and fastening can disrupt exon selection and 3′-end patterns (Fong et al., 2014, 2015; Liu et al., 2017; Aslanzadeh et al., 2018; Muniz et al., 2021; Debès et al., 2023; Welsh and Gardini, 2023; Zukher et al., 2023). We propose that in specific contexts, RNA processing choices are modulated through recruitment of RBPs to sites of transcription initiation, allowing them to subsequently hitchhike on the elongating transcription machinery, and to be released onto nascent RNA during Pol II pausing or other changes in elongation dynamics (Figure 1B). In this process, the Pol II C-Terminal Domain (CTD) or tissue-specific TFs may provide a scaffold for interactions with RBPs.

Finally, the increasingly appreciated ability of RBPs to associate with DNA, and of TFs to bind RNA, raises the question whether the two processes —transcription initiation and RNA processing— are as separately controlled by each of the two protein groups as previously thought. Individual examples of RBPs activating transcription have been reported (Zeng et al., 2016; Bi et al., 2019; Ren et al., 2021; Xu et al., 2023), indicating that tissue-specific regulators of RNA processing may also be involved in the activation of distinct promoters. In light of promoter dominance, the RBP-mediated activation of neuron-specific promoters may constitute one mechanism by which neuronal 3′-ends and, more generally, tissue-specific mRNA isoforms are selected. In this context, it is important that nascent pre-mRNAs represent not only mere products, but also important regulators of transcription and RNA processing (Skalska et al., 2017). RBPs may bind nascent RNAs as early as transcription initiation and influence the transcription process (Figure 1C). Similarly, TFs may be recruited to sites of APA and AS by initially binding nascent RNAs in the vicinity of promoter regions.

## Conclusion

Neuron-specific RNA processing is pervasive and occurs in all animals that have been studied, including humans. Variations in 3′ UTR length and sequence contribute to neurological disorders, emphasizing the importance of alternative mRNA processing in nervous system development and physiology (Mohanan et al., 2021; LaForce et al., 2022; Wilson et al., 2023). It has become more and more evident that cis-regulatory elements and their associated biomolecules —transcription factors, coding and non-coding RNAs— contribute to the generation of neuron-specific exons and 3′ UTRs. Recent advances in long-read RNA sequencing, chromatin capture studies, protein-nucleic acid interaction analyses, and imaging of nascent mRNAs, have already provided glimpses into the coordination of co-and post-transcriptional processes. Systematically applying these approaches to nervous system tissues, in combination, will shed light on the mechanisms that link transcription and RNA processing, and help identifying and possibly targeting disease-causing mutations.

## Author contributions

HO: Conceptualization, Visualization, Writing – original draft, Writing – review & editing. VH: Conceptualization, Visualization, Writing – original draft, Writing – review & editing.
